# Fabrication and Characterization of Viton@FOX-7@Al Spherical Composite with Improved Thermal Decomposition Property and Safety Performance

**DOI:** 10.3390/ma14051093

**Published:** 2021-02-26

**Authors:** Xiaodong Li, Yue Yang, Changgui Song, Yantao Sun, Yuanqi Han, Yue Zhao, Jingyu Wang

**Affiliations:** School of Environment and Safety Engineering, North University of China, Jiancaoping District, Taiyuan 030051, China; song986522760@163.com (C.S.); sunyt@nuc.edu.cn (Y.S.); Cadits@yeah.net (Y.H.); yy080044@126.com (Y.Z.); wjywjy67@163.com (J.W.)

**Keywords:** FOX-7 (1,1-diamino-2,2-dinitroethylene), thermal decomposition analysis, energetic materials, aluminized composite explosives

## Abstract

To achieve a uniform distribution of the components and a better performance of aluminized composite explosives, Viton (dipolymers of hexafluoropropylene and vinylidene fluoride) @ FOX-7 (1,1-diamino-2,2-dinitroethylene) @Al microspheres and FOX-7/Viton@Al were synthesized by spray-drying strategy contrastively. Viton@FOX-7@Al owned porous and loose morphology and good sphericity with a retained crystal phase of FOX-7 and aluminum. The 23.56% fluorine content on Viton@FOX-7@Al surface indicated that Viton was completely coated on the surface of the particles. Nanosized aluminum (nAl) in Viton@FOX-7@Al had a certain catalytic activity on the thermal decomposition process of FOX-7 resulting in a depressed exothermic peak temperature and reduced apparent activation energy relative to nAl in FOX-7/Viton@Al. Because of the specific structure and the synergies between each individual component, Viton@FOX-7@Al showed reduced impact sensitivity and friction sensitivity than those of FOX-7/Viton@Al. In brief, Viton@FOX-7@Al with multilevel coating structure possessed comparatively low thermal decomposition energy requirement and improved safety performance.

## 1. Introduction

Aluminized composite explosives are usually composed of heterogeneous components, including explosive crystals as high-energy components, polymers as binders and aluminum powder as the main metal fuel. Due to the high energy output and explosion heat, aluminized composite explosives are widely used in advanced weapon systems. [1,2,3]. Aluminum powder is used as a fuel additive to increase the energy of the air blast wave, the bubble energy in underwater explosions, and the duration of combustion action in aluminized composite explosives [4,5,6]. Compared to micrometer-sized particles, nano-sized aluminum (nAl) particles own high special surface areas and enhanced contact areas with explosives, so as to achieve more complete and faster oxidation [4,7].

However, because of the inevitable aggregation of nAl and the significant difference in particle size distribution between nAl and explosive particles, the distribution of different components is generally inhomogenous, which greatly limits the performance of aluminized composite explosives [8]. To achieve uniform distribution of the additives, spray-drying as a facile way of assembling heterogeneous components could be applied in the preparation of composite energetic materials [6,9,10]. The spray-drying method was applied to fabricate TATB-wrapped HMX composites with micro-sized HMX particles embedded uniformly into the TATB encapsulation. The results indicated that the spray-drying technique was a facile and preferred strategy for the formation of core-shell energetic composites with high performance [11]. Ji et al. adopted a suspension spray-drying method to prepare solid spherical HMX/F_2602_ possessing good thermal stability and lowered mechanical sensitivity [12], which denoted spray-drying as a promising method for the preparation of the energetic particles with desirable structure.

Herein, the Viton@FOX-7@Al microspheres and FOX-7/Viton@Al were fabricated contrastively by spray-drying through solvent volatilization and the precipitation of FOX-7 and Viton. The morphology and composition of recrystallized FOX-7, nAl, Viton@FOX-7@Al and FOX-7/Viton@Al were measured and analyzed. The thermal properties and mechanical sensitivity of the three samples were further characterized. Our work demonstrated a facile and effective spray-drying method for preparing aluminized composite explosives with controlled structure and improved performance, providing a good combination of technology and material structure.

## 2. Materials and Methods

### 2.1. Sample Preparation

The materials used herein were recrystallized FOX-7 (purity 99.5%), which was prepared from pristine FOX-7 [13] (Modern Chemistry Research Institute of China, Xi’an) in our laboratory, 50–100 nm aluminum powders (nAl, Beijing Flance Nanotechnology Co., Ltd., Beijing, China) and Viton (Bluestar Chengrand Co., Chengdu, China). The reagents used in this study were dimethylformamide (DMF, AR, Tansoole, Shanghai, China) and ethyl acetate (EtAc, AR, Tansoole, Shanghai, China).

The preparation of two different suspensions: 0.20 g Viton was dissolved in 96 mL DMF and EtAc to form two different transparent solutions, respectively. The recrystallized FOX-7 grains (3.00 g) and nAl (0.80 g) were added into the two different Viton solutions, respectively. Two different uniform suspensions were formed after stirring (300 rpm) and sonication (650 W) for 30 min (DMF: nAl suspended in FOX-7/Viton co-solution; EtAc: FOX-7 and nAl suspended in Viton solution) for further preparation of the composites.

The preparation of two different composites: two kinds of aluminized FOX-7 based composites were obtained by spray-drying (Figure S1) under the same experimental conditions (using a feed rate of 4.5 mL·min^−1^, a nitrogen flow rate of 336 L·h^−1^, an inlet temperature of 90 °C and a spray cap with a 1.4 mm hole). A blackish green powder (Viton@FOX-7@Al) and a yellow green powder (FOX-7/Viton@Al) were collected after 30 min.

### 2.2. Characterization

The characterization of the composition, morphology and crystalline structures of the three samples was performed by field-emission scanning electron microscopy (FE-SEM; S4800, Hitachi, Tokyo, Japan), transmission electron microscope (TEM, JEOL JEM-2100F, Tokyo, Japan), X-ray powder diffraction (XRD; DX-2700 Dandong Haoyuan, using Cu Ka radiation, Dandong, Liaoning, PR China) and X-ray photo electron spectroscopy (XPS; Thermo ESCALAB 250Xi X-ray photoelectron spectrometer, Carlsbad, CA, USA), respectively. The program Nano Measurer 1.2 was utilized for size measurement. Differential scanning calorimetry (DSC) was characterized on a TA Q2000 instrument (TA Instruments, NewCastle, DE USA) at the heating rate of 5, 10, 15, and 20 °C·min^−1^ ranging from 50 °C to 350 °C under air atmosphere. The impact sensitivity and friction sensitivity of the three samples were tested according to the Chinese National Military Standard (GJB-772A-97) [14] method 601.3 with an ERL Type 12 drop-hammer instrument and the GJB-772A-97 method 602.1 with a WM-type friction sensitivity apparatus, respectively (See Figures S4 and S5).

## 3. Results

### 3.1. XRD Analysis

**Figure 1:**
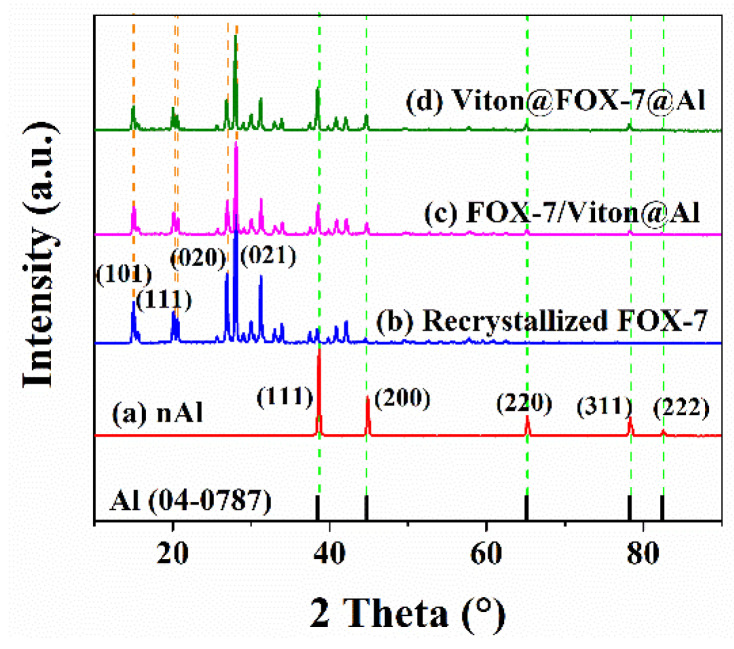
XRD patterns of (**a**) nAl; (**b**) recrystallized FOX-7; (**c**) FOX-7/Viton@Al and (**d**) Viton@FOX-7@Al. The XRD patterns were measured within the 2θ range of 10°~90° at a scan speed of 0.5°·min^−1^.

XRD was utilized in detecting the phase composition of the prepared samples. Figure 1 depicts the XRD patterns of nAl, recrystallized FOX-7, FOX-7/Viton@Al and Viton@FOX-7@Al. All of the diffraction peaks of nAl were perfectly matched to the aluminum (JCPDS No. 04-0787) with no aluminum oxide peaks found [15]. Meanwhile, FOX-7/Viton@Al and Viton@FOX-7@Al both showed diffraction peaks of FOX-7 crystal and nAl and the significantly weakened diffraction peak intensity of nAl indicated that the nAl surface had been completely coated in two different structured composites.

### 3.2. XPS Analysis

**Figure 2:**
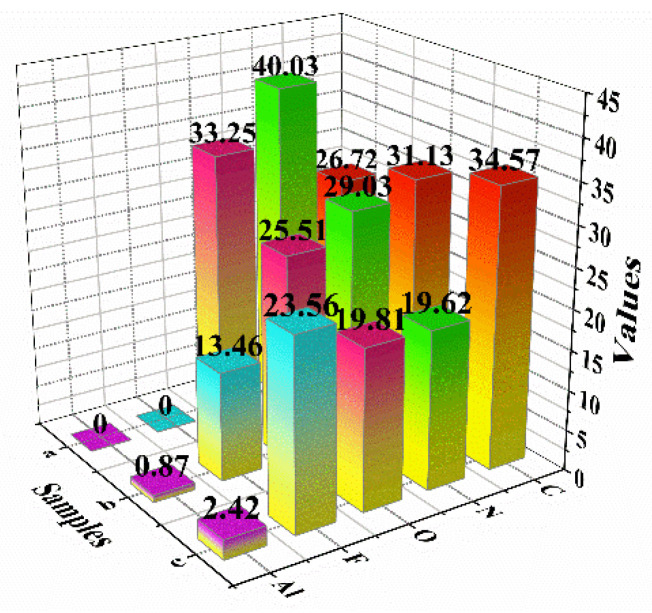
Quantification of element species on the surface of the three samples through analysis of XPS: (**a**) recrystallized FOX-7, (**b**) FOX-7/Viton@Al and (**c**) Viton@FOX-7@Al.

The XPS tests were carried out to illustrate the surface elemental composition and chemical state of the three samples, namely recrystallized FOX-7, FOX-7/Viton@Al and Viton@FOX-7@Al. The survey spectra showed that the major elements on the surface of recrystallized FOX-7 were C, N and O and the major elements on the surface of FOX-7/Viton@Al and Viton@FOX-7@Al were C, N, O, F and Al (Figure 2). The intensity of N and O element content in the two coated samples was apparently reduced, indicating a depressed amount of FOX-7 on the surface of the two composites. The element content of Al on the surface of FOX-7/Viton@Al and Viton@FOX-7@Al was 0.87% and 2.42% respectively, which denotes good coating effect of Viton and FOX-7 on nAl surface. The content of fluorine on the surface of the Viton@FOX-7@Al was relatively high, while the content of oxygen and nitrogen on the surface of the Viton@FOX-7@Al was reduced, indicating that the Viton was mainly distributed and coated on the surface of Viton@FOX-7@Al although the content of Viton in the precursor was lower than that of FOX-7.

**Figure 3:**
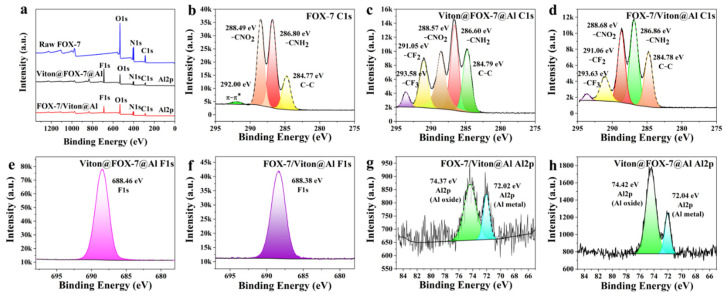
(**a**) XPS survey spectra of the three samples; The detailed spectra of the C1s peaks for (**b**) recrystallized FOX-7; (**c**) FOX-7/Viton@Al and (**d**) Viton@FOX-7@Al; the detailed spectra of the F1s peaks for (**e**) FOX-7/Viton@Al and (**f**) Viton@FOX-7@Al; the detailed spectra of the Al2p peaks for (**g**) FOX-7/Viton@Al and (**h**) Viton@FOX-7@Al.

The C1s peaks of recrystallized FOX-7 appeared at 284.77 (C–C), 286.80 (C–NH_2_), 288.49 (C–NO_2_), 292.00 (π–π* molecular transition) (Figure 3b), which is in agreement with the crystal structure of FOX-7 in reference [16]. Compared with recrystallized FOX-7, the peaks of FOX-7/Viton@Al and Viton@FOX-7@Al assigned to π–π* molecular transition were completely covered by two appeared peaks corresponding to –CF_2_ and –CF_3_, which confirmed that Viton was incorporated into two spray-dried composites respectively. The intensity of F1s peak of Viton@FOX-7@Al was apparently higher than that of FOX-7/Viton@Al, indicating a higher coverage of Viton on the Viton@FOX-7@Al surface. The detailed spectra over the Al2p peaks for FOX-7/Viton@Al and Viton@FOX-7@Al are shown in Figure 3g,h. The spectra could be fitted as a high binding energy peak and a low shoulder peak [17], corresponding to Al in some oxidized form and metal Al in the core of composites, respectively. Evidently, the relative intensity for Al2p in FOX-7/Viton@Al was lower, which indicated the coating of Viton on nAl without covering the surface of FOX-7.

### 3.3. Morphology Analysis

**Figure 4:**
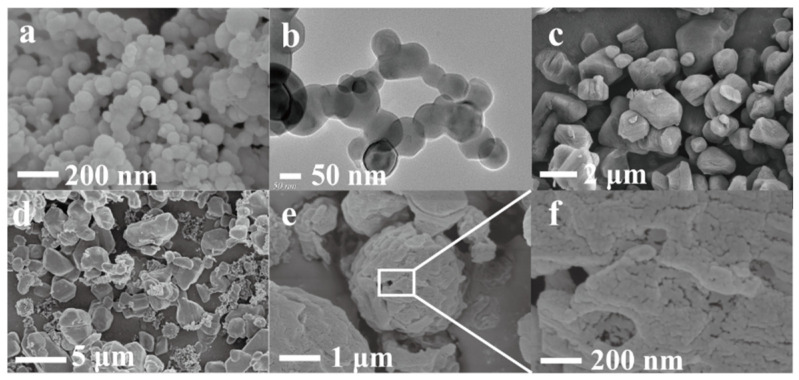
(**a**) SEM images of nAl, (**b**) TEM image of nAl, SEM images of (**c**) recrystallized FOX-7, (**d**) FOX-7/Viton@Al, (**e**,**f**) Viton@FOX-7@Al.

As shown in Figure 4, the morphology of nAl, recrystallized FOX-7, FOX-7/Viton@Al and Viton@FOX-7@Al were obtained by SEM measurement. The perfect spherical shape of nAl could be observed in Figure 4a. As shown in Figure 4b and Figure S2, the oxide thickness of nAl was about 1~2 nm, indicating good reactive activity of nAl. The recrystallized FOX-7 grains are shown in Figure 4c, displaying cubic crystals with the particle size centralized in the range of 0.63 ~ 2.22 μm. The morphology of FOX-7/Viton@Al and Viton@FOX-7@Al composites obtained by the spray-drying process are shown in Figure 4d,f, respectively. As shown in Figure 4d, the surface of FOX-7 grains in FOX-7/Viton@Al was almost bare without the covering from Viton, which was in conformity with the aforementioned XPS results. The particle size of FOX-7/Viton@Al was the same as that of recrystallized FOX-7, while Viton was mainly coated on the surface of nAl. Contrastingly, Viton@FOX-7@Al was loose and porous microspheres with a particle size of about 3 μm showing a completely different morphology from that of recrystallized FOX-7 (Figure S3).

**Figure 5:**
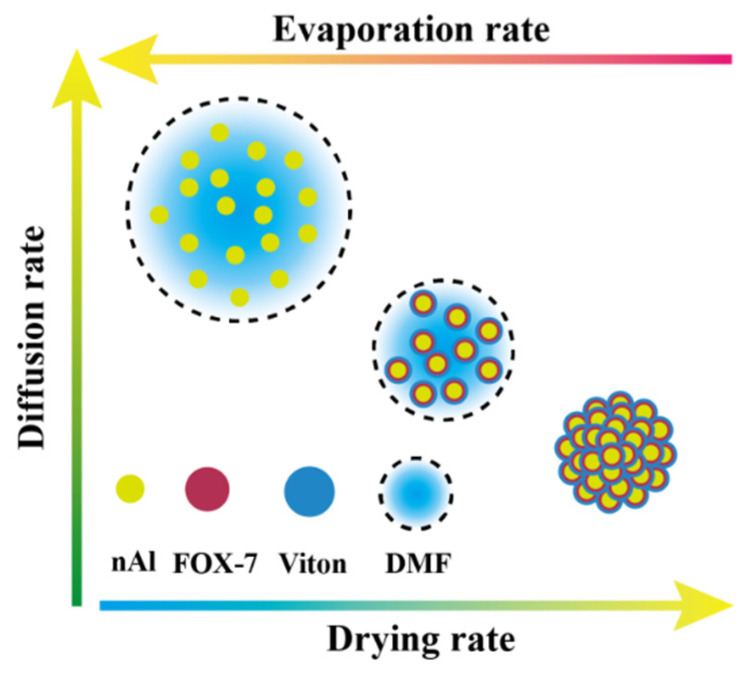
Schematic diagram showing the development of a droplet consisting of the co-solution of FOX-7 and Viton dispersed nAl during the spray-drying process.

The reason for the appearance of the loose and porous composite spheres of Viton@FOX-7@Al could be as explained below (Figure 5). First off, the co-solution of Viton and FOX-7 dispersed with nAl spheres was atomized into droplets and entered the drying chamber at 90 °C. The DMF on the droplet surface was rapidly evaporated with the heating of drying nitrogen. The condensation of the FOX-7 and Viton formed a dried shell outside the droplets, which reduced the possibility of further adhesion among the droplets [18,19]. Inevitably, the inner DMF carrying solutes was diffused to the surface [20]. The vapor pressure inside the particles increased due to the vaporization of DMF under heating condition. One or more gaps gradually formed in the shell because the surface enrichment of the particles prevented the inner high-pressure steam from dissipating immediately. Meanwhile, components of Viton and FOX-7 wrapped nAl particles were solidified down to constitute a thicker shell. Thus, the loose and porous, energetic particles with a certain number of gaps were constructed by a facile spray-drying strategy.

### 3.4. DSC Analysis

**Figure 6:**
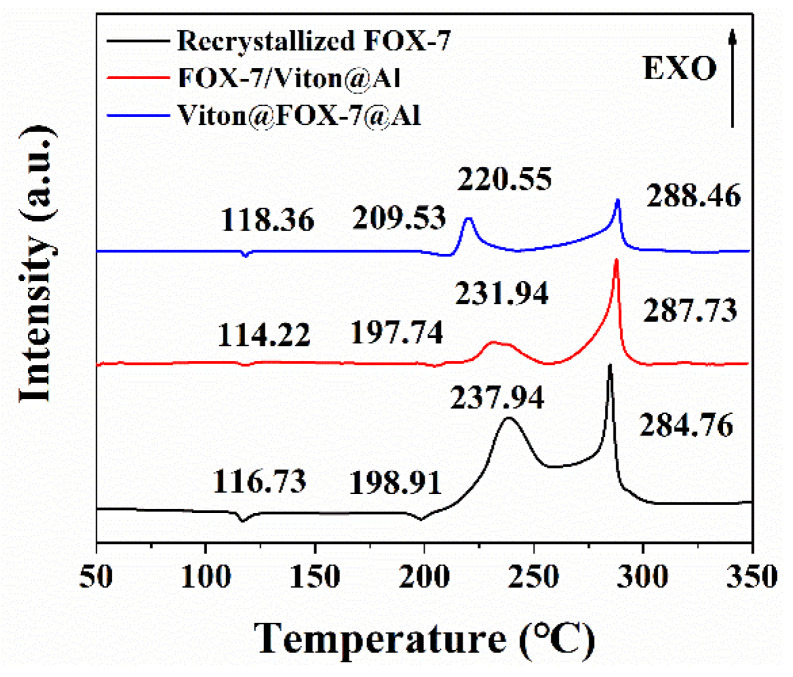
DSC curves of recrystallized FOX-7, FOX-7/Viton@Al and Viton@FOX-7@Al under the heating rate of 5 °C·min^−1^.

The thermal decomposition behaviors were obtained according to the DSC curves of three different samples at 5 °C·min^−1^. As shown in Figure 6, three different samples had a similar exothermic decomposition process, which indicated that solid-phase decomposition kinetics dominated their thermal decomposition process. The DSC curves showed the first endothermic peak for the β → γ phase transition [21] and the second endothermic peak for the γ → δ transition [22]. After the phase transitions, there were two successive exothermic decomposition processes of FOX-7: the first with the peak at ca. 230 °C corresponding to the emergence of nitro-to-nitrite rearrangement and the second with a main exothermic peak at ca. 290 °C attributing to the break of the carbon skeleton. Compared with the exothermic peak temperature (*T_p_*) of recrystallized FOX-7 grains, the *T_p_* values of the two FOX-7/Al based composites decreased by 6.00 and 17.39 °C, respectively, showing the beneficial effect of nAl in accelerating the thermal degradation of the FOX-7 molecule. Because of the close interaction between FOX-7 and nAl in multilevel structured Viton@FOX-7@Al, the promotion effect of nAl on the energy release process of FOX-7 in Viton@FOX-7@Al was more obvious than that of FOX-7/Viton@Al.

**Figure 7:**
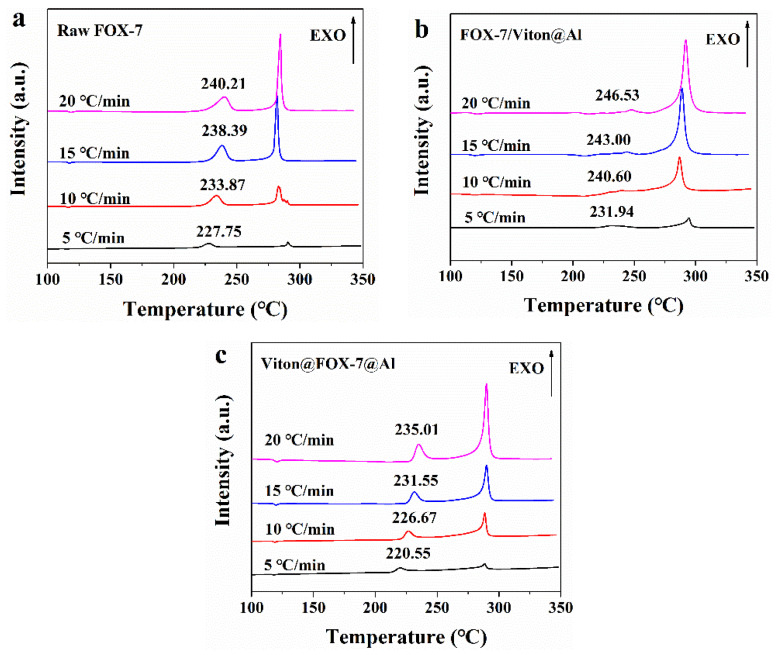
DSC patterns of (**a**) recrystallized FOX-7, (**b**) FOX-7/Viton@Al and (**c**) Viton@FOX-7@Al under the heating rates of 5, 10, 15 and 20 °C·min^−1^.

To achieve the effective evaluation and better functionality of the composite structure formed by FOX-7, nAl and Viton, it was necessary to estimate the kinetic parameters [23]. We measured *T_p_* at different heating rates of 5, 10, 15, and 20 °C·min^−1^ (Figure 7) and presented averaged apparent activation energy (*E_α_*) values calculated by Kissinger, Starink and Ozawa methods [24,25,26]. The natural logarithmic value of preexponential factor (*ln A*) was obtained from *E_α_* value calculated by Kissinger method for the first decomposition stage (See Supplementary Materials) and was listed in Table 1.

It is worth noting that the averaged *E_α_* value of the initial thermal decomposition process in two composite samples was much lower compared to that of recrystallized FOX-7 crystal, which signified nAl had a certain catalytic activity on the thermal degradation of FOX-7 grains. Thus, the decomposition of FOX-7 in the two composites occurred at a lower temperature (with minimum energy requirement). Due to the huge specific area and high surface free energy, nAl tended to absorb gaseous reactive molecules and promote the decomposition reactions of FOX-7 with high catalytic activity [1,4].

The linear relationship between *ln A* and *E_α_* is considered as the kinetic compensation effect which is widely existed in kinetic studies of solid-state reactions [27]. The values of linear correlation coefficients *r^2^* lay between 0.9678 to 0.9980, which were high enough to represent a good fitting degree of the methods. The values of *E_α_* obtained by the three methods were similar, proving that the three methods were appropriate for activation energy threshold calculations. The reduced critical temperature of thermal explosion (*T_b_*) values in Viton@FOX-7@Al and FOX-7/Viton@Al indicated the catalytic effect of nAl on energy release process of the FOX-7 grains.

The analysis of thermodynamic parameters is also an important part along with kinetics for the effectively evaluation of the thermal decomposition properties because these parameters provide fundamental information about the difficulty of activation reaction and thermodynamic equilibrium during the thermal decomposition processes of the samples [28,29]. The calculated results of thermodynamics are listed in Table 2.

Molecular disorder and randomness of the system are defined by entropy (Δ*S^≠^*). The decreased Δ*S^≠^* value indicated a lower degree of disorder of the products compared to that of recrystallized FOX-7. The positive nature of enthalpy (Δ*H^≠^*) denoted the requirement of heat supplement during thermal decomposition of the samples. Gibbs free energy (Δ*G^≠^*) is a critical thermodynamic property, which signified the available chemical energy that could be applied to convert reactants into the activated substances. Compared with recrystallized FOX-7, the barely unchanged values of Δ*G^≠^* of the composites revealed that the three samples had sufficient energy to be transited into final state.

### 3.5. Mechanical Sensitivity

The impact sensitivity and friction sensitivity tests of the three different samples were carried out. Table 3 shows the results of sensitivity analysis, reflecting as a 50% reaction level (*H_50_*) in impact sensitivity and the explosion probability in the friction sensitivity test.

The *H*_50_ values of FOX-7/Viton@Al and Viton@FOX-7@Al were improved from 128.3 cm (recrystallized FOX-7) to 135.4 cm and > 160 cm, respectively, and the friction sensitivity of FOX-7/Viton@Al and Viton@FOX-7@Al were reduced from 19% (recrystallized FOX-7) to 12% and 0%, respectively (Table 3). The changed values of *H*_50_ and friction sensitivity indicated that Viton@FOX-7@Al had a better safety performance although FOX-7/Viton@Al and Viton@FOX-7@Al were composed of the same components.

The mechanism of the mechanical sensitivity reduction of FOX-7/Viton@Al and Viton@FOX-7@Al could be explained as follows. The high thermal conductivity caused by the addition of nAl led to a fast heat transfer process in the composites under external stimulation, which could decrease the probability of heat centralization and further reduce hot-spot generation [30,31]. Besides, Viton as the cladding material formed a buffer layer on the surface of FOX-7 and nAl, decreasing the external stimuli impacted on the surface of FOX-7 and nAl [32]. Moreover, Viton@FOX-7@Al had a multilevel structure integrated by Viton and FOX-7, providing a higher surface area of FOX-7 than that of FOX-7 in FOX-7/Viton@Al. Compared with FOX-7/Viton@Al, the high surface area of FOX-7 and close contact between FOX-7 and nAl accelerated the heat transfer rate in Viton@FOX-7@Al when suffering from rapid and intensive mechanical impact and friction.

## 4. Conclusions

To sum up, FOX-7/Viton@Al and Viton@FOX-7@Al with different structures were successfully synthesized by the spray-drying method. Viton@FOX-7@Al with loose and porous spherical shape had a higher fluorine content on the surface, which indicated that the surface of nAl was fully coated. The reduced values of apparent activation energy and exothermic peak maximum showed that the catalytic effect of nAl in Viton@FOX-7@Al was superior to that in FOX-7/Viton@Al. Viton@FOX-7@Al had lower impact and friction sensitivity relative to FOX-7/Viton@Al, which confirmed that multilevel structured Viton@FOX-7@Al had higher safety performance. This study shows that Viton@FOX-7@Al has considerable potential for further application in the field of composite solid propellant.

## Figures and Tables

**Table 1:** Thermal decomposition kinetics of samples.

Samples	*E_a_* (kJ·mol^−1^)			*Averaged E_a_* (kJ·mol^−1^)	*ln A (*s^−1^)	*r^2^*
	Kissinger	Starink	Ozawa			
Recrystallized FOX-7	294.56	303.13	295.42	297.70	68.87	0.9678
FOX-7/Viton@Al	200.40	208.91	201.25	203.52	46.95	0.9980
Viton@FOX-7@Al	190.75	199.08	191.59	193.81	45.75	0.9914

**Table 2:** Critical temperature of thermal explosion (*T_b_*) and thermal decomposition dynamics of samples.

Samples	*T*_0_ (°C)	*T_b_* (°C)	Δ*S^≠^*/(J·mol^−1^·K^−1^)	Δ*H^≠^*/(kJ·mol^−1^)	Δ*G^≠^*/(kJ·mol^−1^)
Recrystallized FOX-7	223.55	230.64	315.25	290.43	133.84
FOX-7/Viton@Al	216.63	224.55	133.03	196.38	132.16
Viton@FOX-7@Al	213.37	223.97	123.11	186.71	126.81

**Table 3:** Impact sensitivity and friction sensitivity of the samples.

Samples	Impact Sensitivity *H*_50_ (cm)	Friction Sensitivity (%)
Recrystallized FOX-7	128.3	19
FOX-7/Viton@Al	135.4	12
Viton@FOX-7@Al	>160	0

## Data Availability

The data presented in this study are available in supplementary material.

## Supplementary Materials

The following are available online at https://www.mdpi.com/1996-1944/14/5/1093/s1, Figure S1: Schematic diagram of spray-drying device. Figure S2 (**a**) TEM image of nAl, (**b**) the oxide thickness of nAl from the TEM image. Figure S3 (**a**) SEM image of Viton@FOX-7@Al, (**b**) the particle size distribution of Viton@FOX-7@Al from the SEM image. Figure S4: Illustration of HGZ-1 impact instrument. Figure S5: Experimental set-up for measuring friction sensitivity.
