# Supplementary material for: Fabrication and Characterization of Viton@FOX-7@Al Spherical Composite with Improved Thermal Decomposition Property and Safety Performance
Source: Materials (Basel). 2021 Feb 26;14(5):1093. doi: 10.3390/ma14051093 (PMC7956709; doi:10.3390/ma14051093)
Supplement: Supplementary file 1 [file materials-14-01093-s001.pdf]

coatings

Supplementary Materials: Fabrication and Characterization of Viton@FOX-7@Al Spherical Composite with Improved Thermal Decomposition Property and Safety Performance

Xiaodong Li *, Yue Yang *, Changgui Song, Yantao Sun, Yuanqi Han, Yue Zhao and Jingyu Wang

School of Environment and Safety Engineering, North University of China, Jiancaoping District, Taiyuan 030051, China; song986522760@163.com (C.S.); sunyt@nuc.edu.cn (Y.S.); Cadits@yeah.net (Y.H.); yy080044@126.com (Y.Z.); wjywjy67@163.com (J.W.)

* Correspondence: lixd78@126.com (X.L.) and yy.amie@foxmail.com (Y.Y.).

Citation: Li, X.; Yang, Y.; Song, C.; Sun, Y.; Han, Y.; Zhao, Y.; Wang, J. Fabrication and Characterization of Viton@FOX-7@Al spherical composite with improved thermal decomposition property and safety performance. *Materials* **2021**, *13*, 1093. https://doi.org/10.3390/ma14051093

Academic Editor: Gabriele Milani

Received: 5 January 2021
Accepted: 11 February 2021
Published: 26 February 2021

Publisher's Note: MDPI stays neutral with regard to jurisdictional claims in published maps and institu-tional affiliations.

Copyright: © 2021 by the authors. Licensee MDPI, Basel, Switzerland. This article is an open access article distributed under the terms and conditions of the Creative Commons Attribution (CC BY) license (http://creativecommons.org/licenses/by/4.0/).

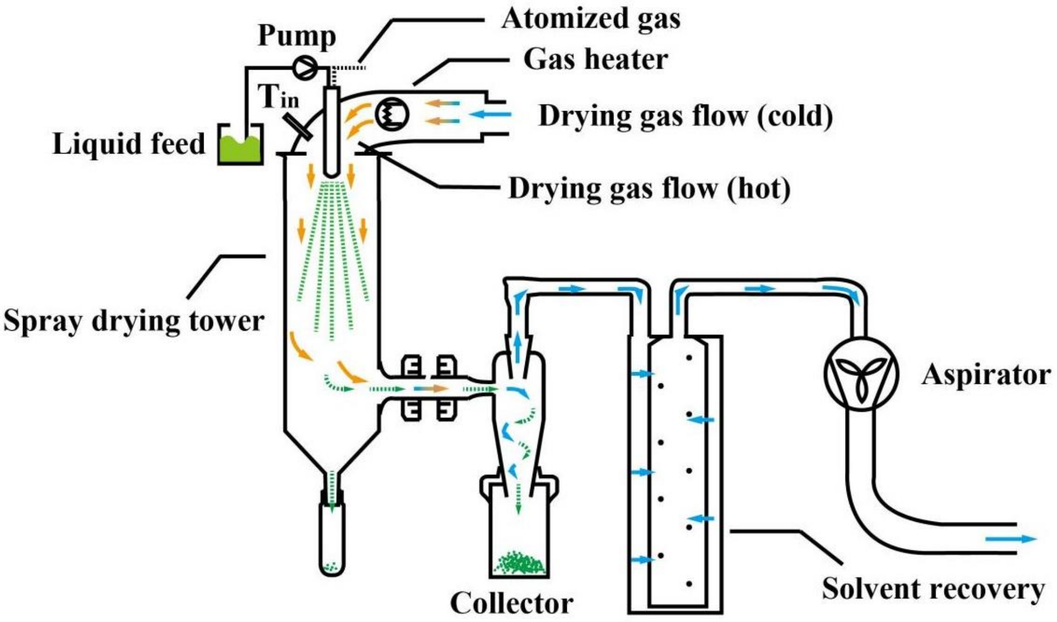

Figure S1. Schematic diagram of spray drying device.

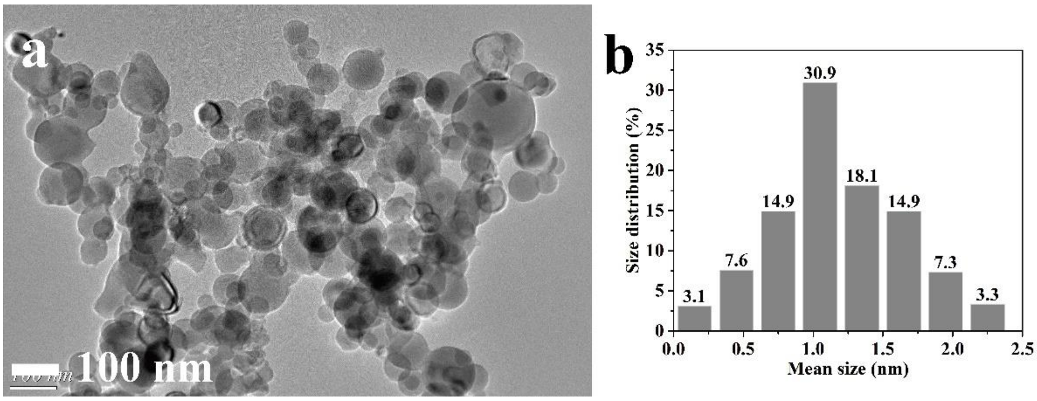

Figure S2. (a) TEM image of nAl, **(b)** the oxide thickness of *n*Al from the TEM image.

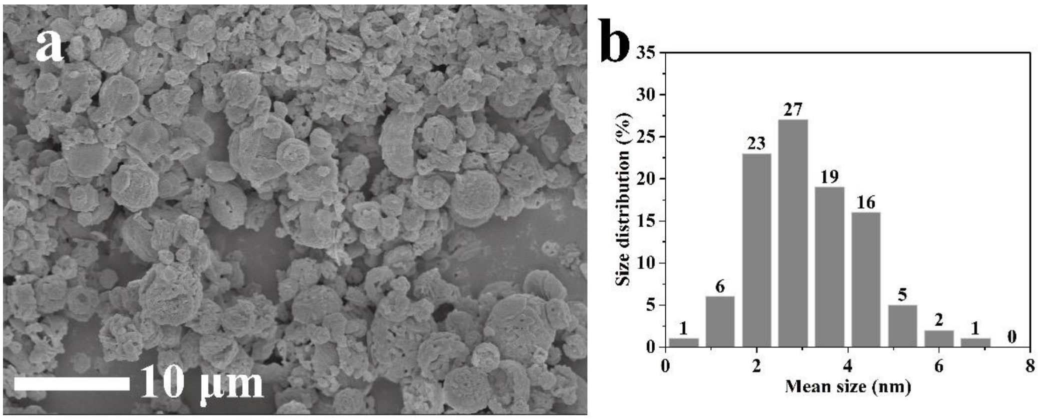

Figure S3. (a) SEM image of Viton@FOX-7@Al, **(b)** the particle size distribution of Viton@FOX-7@Al from the SEM image.

Thermal decomposition analysis

Non-isothermal kinetics analysis

To get the kinetic information such as activation energy, with different heating rates kinetic study was conducted. To get the activation energy (E_α) and pre-exponential factor (A) from DSC analysis was calculated according to Ozawa, Kissinger and Starink methods. These reported methods are widely used for various kinetic parameters determination. The activation energy obtained and determined by Kissinger, Ozawa, and Starink methods according to the equations No. (1) (2) and (3) respectively.

Kissinger Method

The Kissinger method can be expressed by the following equation:

$$\ln(\beta / T_p^2) = ln(AR/E_\alpha g(\alpha)) - E_\alpha / RT_p \quad (1)$$

Where β is the heating rate. The $g(\alpha)$ is the mechanism and can be taken as unity and it is constant at a particular value of the conversion. The α indicates the value of the conversion and R the general constant. Through this method we can calculate the E_α by plotting $ln(\beta/Tp2)$ vs. $1000/T$, and we can calculate the slope from $-E_\alpha/R$.

Ozawa Method

The Ozawa method simple methematical form can be expressed as:

$$\ln(\beta / T_p^2) = ln(AR/E_\alpha g(\alpha)) - E_\alpha / RT_p \quad (2)$$

Where E_α is the activation energy, The plot of $(ln(\beta))$ vs $1000/T$ gives the slope which is equal to ($-1.052E_\alpha/R$). E_α can be calculated at different heating rates of the given slope.

Starink Method

In 1996, a novel method was developed by Starink, combining the Kissinger and Ozawa methods, Kissinger and Ozawa methods can be formulated as following.

$$\ln(\beta / T_p^{1.92}) = Constant - 0.312 - (1.0008 E_\alpha / RT_p) \quad (3)$$

In the above mentioned technique, if we plot [$ln(\beta/T_p^{1.92})$], the slope would gives us E_α. The pre-exponential factor (A) was calculated by the following equation according to Kissinger method:

$$A = [\beta \cdot E_\alpha \exp(E_\alpha / RT_P)]/(RT_P^{\,2}) \quad (4)$$

Critical temperature of thermal explosion

The critical temperature of thermal explosion (T_b) is an important indicator for the thermal safety of energetic materials during storage and operation, Therefore, based on the value of the peak temperature corresponding to $\beta \rightarrow 0$ (T_0) obtained from the equations No. (5), equations No. (6) are applied to determine the values of T_b.

$$T_0 = T_p - (a\beta + b\beta^2 + c\beta^3) \quad (5)$$

$$T_b = (E_\alpha - \sqrt{E_\alpha^{\;2} - 4E_\alpha RT_0})/2R \quad (6)$$

Where a, b and c are coefficients.

Thermodynamic study

After estimating the activation energy by three methods at four different heating rates, the thermodynamic properties like enthalpy ($\Delta H^{\neq}$), Gibbs free energy ($\Delta G^{\neq}$) and entropy ($\Delta S^{\neq}$) were evaluated by using subsequent equations below:

$$\Delta H^{\neq} = E_a - RT_0 \quad (7)$$

$$\Delta G^{\neq} = E_\alpha + RT_p ln[(K_B / T_p)/(hA)] \quad (8)$$

$$\Delta S^{\neq} = (\Delta H^{\neq} - \Delta G^{\neq}) / T_p \quad (9)$$

Here, T_p represents the peak temperature from DSC curve at particular heating rate. Boltzmann constant with a value of 1.381×10^{-23} J/K is represent by K_B, and h donates the plank constant (6.626×10^{-34} J s).

Impact sensitivity

The procedure was as follows: the weighed sample flowed out through a cone funnel at a constant speed and piled up on the center of the sandpaper to form a pyramid. The sandpaper with the sample was then carefully placed at the center of the anvil and the impact plunger was gently pressed against the sample. The power supply was turned on, the lift button pressed and the desired height of the ball seat adjusted (the height can be obtained from the flexible rule). The pump button was then turned on and the ball seat was pumped into a vacuum. The dropping hammer was adsorbed at the desired height, when it was near the ball seat. The pump button was turned off. The dropping hammer then became free and started to free fall until it hit the impact plunger. Then the sample on the sandpaper was impacted to explode by the impact plunger. The hammer anvil was used to buffer the residual impact after test. When the dropping hammer fell from the setting height, the sample exploded. This setting height is the special height H_{50}. The samples were tested for impact sensitivity using HGZ-1 impact instrument, whose structure diagram is shown in Fig. S4.

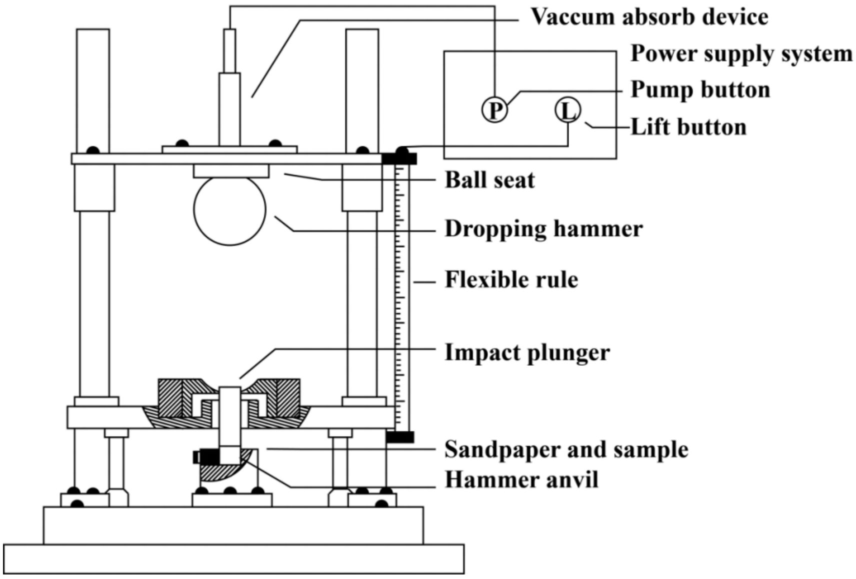

Figure S4. Illustration of HGZ-1 impact instrument.

Friction sensitivity

The friction sensitivity test was conducted as follows: the weighed sample was carefully placed between two slip sleeves and the clamp sleeve tightened. The pressure-biased valve was turned on and the pressured adjusted to 3.92 MPa. The hammer anvil was inserted and checked that it touched the clamp sleeve. The pendulum was lifted and then released at a swaying angle of 90 deg. Finally, the hammer anvil was taken out and the pressure relief valve was turned on. The friction sensitivity test was completed. Then

the explosion probability of the sample was obtained by calculating the proportion of the total number of explosions to the total number of experiments.

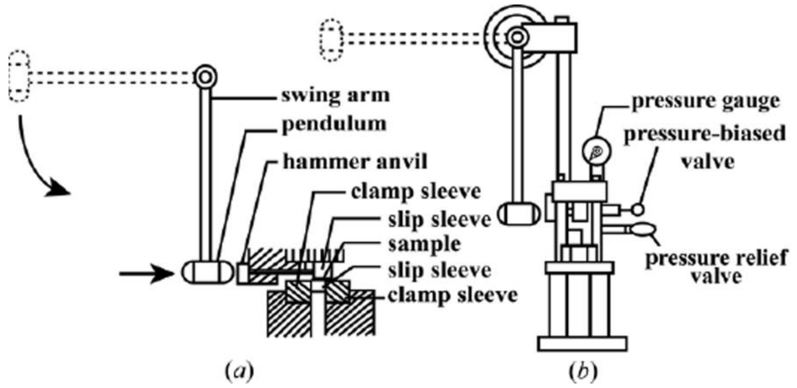

Figure S5. Experimental set-up for measuring friction sensitivity.
